# Eculizumab prevents thrombotic microangiopathy in patients with atypical haemolytic uraemic syndrome in a long-term observational study

**DOI:** 10.1093/ckj/sfy035

**Published:** 2018-05-16

**Authors:** Jan Menne, Yahsou Delmas, Fadi Fakhouri, John F Kincaid, Christoph Licht, Enrico E Minetti, Chris Mix, François Provôt, Eric Rondeau, Neil S Sheerin, Jimmy Wang, Laurent E Weekers, Larry A Greenbaum

**Affiliations:** 1 Klinik für Nieren- und Hochdruckerkrankungen, Hannover, Germany; 2 CHU de Bordeaux, Bordeaux, France; 3 CHU de Nantes, Nantes, France; 4 Alexion Pharmaceuticals, Inc., New Haven, CT, USA; 5 The Hospital for Sick Children, Toronto, Ontario, Canada; 6 Azienda Ospedaliero Universitaria Careggi, Florence, Italy; 7 CHU de Lille, Lille, France; 8 Hôpital Tenon and Université Paris VI, Paris, France; 9 Institute of Cellular Medicine, University of Newcastle upon Tyne, Newcastle upon Tyne, UK; 10 CHU de Liège, Liège, Belgium; 11 Emory University School of Medicine and Children’s Healthcare of Atlanta, Atlanta, GA, USA

**Keywords:** atypical haemolytic uraemic syndrome, complement, discontinuation, eculizumab, observational study, thrombotic microangiopathy

## Abstract

**Background:**

Eculizumab, a terminal complement inhibitor, is approved for atypical haemolytic uraemic syndrome (aHUS) to inhibit complement-mediated thrombotic microangiopathy (TMA).

**Methods:**

In five parent studies, eculizumab effectively prevented TMA and improved renal and haematologic outcomes in patients with aHUS; therefore, these patients could enrol in this long-term, prospective, observational and multicentre study. The primary endpoint was the TMA manifestation rate off and on eculizumab post-parent study. *Post hoc* analyses evaluated rates during labelled versus non-labelled dosing regimens, and in those with versus without identified complement abnormalities. Serious targeted treatment-emergent adverse events (TEAEs) were evaluated.

**Results:**

Of 87 patients in the current study, 39 and 76 had off- and on-treatment periods, respectively; 17 (44%) with off periods reinitiated eculizumab. TMA manifestation rate per 100 patient-years was 19.9 off and 7.3 on treatment [hazard ratio (HR), 4.7; P = 0.0008]; rates were highest off treatment and lowest during labelled regimens. TMA manifestations with hospitalizations/serious AEs occurred more frequently off versus on treatment. TMA rates were higher among patients with identified complement abnormalities (HR, 4.5; P = 0.0082). Serious targeted TEAEs occurred at similar rates off and on treatment.

**Conclusions:**

As expected, patients with aHUS have increased risk of TMA manifestations after discontinuation of eculizumab or in the setting of non-labelled eculizumab dosing. Collectively, results show that maintaining eculizumab treatment minimizes risk of TMA, particularly in patients with identified complement abnormalities. Future studies are needed to further characterize TMA and longer term outcomes on labelled or non-labelled eculizumab regimens and after discontinuation of treatment.

## INTRODUCTION

Atypical haemolytic uraemic syndrome (aHUS) is a rare, genetic, potentially life-threatening disease predominantly caused by uncontrolled activation of the alternative complement pathway [1–3]. Abnormalities in complement genes or autoantibodies to complement proteins are identified in ≈50–70% of patients [2, 3]. Complement dysregulation leads to persistent cleavage of C5 to the prothrombotic, pro-inflammatory anaphylatoxin C5a and to C5b, which initiates formation of the prothrombotic and cytolytic C5b-9 and ultimately causes injury, activation and lysis of endothelial cells, leucocytes and platelets [1, 4]. The resultant thrombotic microangiopathy (TMA) is typically characterized by microangiopathic haemolytic anaemia, thrombocytopaenia and acute renal failure, and frequently includes extrarenal complications [2, 3].

Patients who remain untreated are at lifelong risk of renal impairment or failure, organ dysfunction and premature death [2, 3]. Eculizumab (Soliris^®^, Alexion Pharmaceuticals, Inc., New Haven, CT, USA), a humanized monoclonal antibody that inhibits C5a, C5b and C5b-9 formation by binding to C5, is the first and only approved treatment for patients with aHUS [5, 6]. The efficacy and safety of eculizumab have been demonstrated in four prospective, multicentre clinical studies [7–10] and a retrospective study [11].

Current regulatory guidance notes potential risk of TMA following discontinuation of eculizumab [5, 6]. Additional evidence for TMA manifestations occurring after discontinuation is limited to case studies [12–25], two retrospective studies [26, 27] and a small prospective observational study [28, 29]. Together, TMA manifestations were documented in 26/82 patients (32%) who discontinued eculizumab [12–29]. An analysis [30] from the eculizumab clinical trial programme determined that severe TMA manifestations occurred in 12/61 patients (20%) who chose to discontinue treatment.

This is the single largest prospective, observational study of the consequences following eculizumab discontinuation in aHUS. In an interim analysis, TMA manifestation rates off and on eculizumab in patients with aHUS were evaluated. *Post hoc* analyses based on a revised, more stringent definition of TMA, during labelled versus non-labelled regimens and by complement abnormality status, also were conducted. In addition, the safety of long-term eculizumab is reported.

## MATERIALS AND METHODS

### Study design and patients

This is a long-term, prospective, observational and multicentre study (NCT01522170) of patients with aHUS who were treated with eculizumab in any of five previous clinical studies (parent studies): four prospective studies (NCT00844545/NCT00844844 and NCT00838513/NCT00844428 [7, 8], NCT01193348 [9], NCT01194973 [10]) and one retrospective study (NCT01770951 [11]). Patients who participated in a parent study were eligible for the current study, regardless of whether they completed or discontinued from the parent study or were on eculizumab at the time of enrolment. Patients could withdraw from the current study at any time. The protocol was approved by an institutional review board or independent ethics committee at each participating centre and the study was conducted in accordance with International Council for Harmonisation Guidelines and the Declaration of Helsinki. All patients and/or parents/guardians provided written informed consent before entry into the current study.

The current study consequently includes both prospective and retrospective data collection. Retrospective data were obtained from the date each patient ended participation in the parent study until the date of signed informed consent for the current study. All patients who received at least one infusion of eculizumab during the parent study and had signed consent forms for the current study were included in the analysis. Identification of complement abnormalities occurred during the parent studies and included analysis of complement factor I (*CFI*), complement factor B (*CFB*), complement factor H (*CFH*), membrane cofactor protein (*MCP*) and *C3* mutations, complement factor H-related proteins 1-3 (*CFHR1-3*) deletions/polymorphisms and CFH autoantibodies [7, 9–11]. Patients received meningococcal vaccination in the parent studies [7, 9, 10] and were revaccinated according to country guidelines. The cut-off date for this interim analysis was 28 March 2015.

TMA manifestations were neither defined nor collected uniformly in the parent studies; thus, this analysis includes outcomes reported in this ongoing observational study only (i.e. beginning at the end of the parent study). Data were collected four times annually in both the retrospective and prospective portions of the current study.

### Treatment

After completion of the parent study and entry into the current study, the labelled dosing regimen of eculizumab was defined as that specified in the prescribing information approved by regulatory authorities [5, 6], and other dosing schedules (i.e. decreased or increased dosages, shortened or extended dosing intervals) were permitted and classified as non-labelled regimens. In this study, the first on-treatment period was defined as: from the date of the first infusion in the current study (i.e. beginning at the end of the parent study) through 3 weeks after the last infusion of eculizumab, or until the patient discontinued from the study, or data cut-off (whichever occurred first). The first off-treatment period was defined as: from 3 weeks after the last infusion of eculizumab within the current study until the patient restarted eculizumab therapy, discontinued from the study or data cut-off (whichever occurred first). Subsequent on- and off-treatment periods were defined similarly. Patient groups were not mutually exclusive; individual patients could be represented in both groups.

### Endpoints

Primary endpoint was the rate of TMA manifestations (defined in Table 1) in the current study off and on treatment. *Post hoc* analyses evaluated TMA manifestation rates off and on treatment when TMA manifestations based on only a single laboratory value change were excluded; in patients receiving labelled compared with non-labelled eculizumab regimens; and in patients with and without identified complement abnormalities. Safety endpoints included assessment of serious targeted treatment-emergent adverse events (TEAEs; predefined as incidence of serious infection, meningococcal infection, sepsis, renal impairment or leucopaenia), as well as any serious AE (SAE).

**Table 1.** Per-protocol definition of TMA manifestations (any ≥1 listed criteria)

Type/severity	Criteria
Laboratory value change only^a^	The occurrence of a change in ≥1 laboratory value^b^:
	Platelet count decrease ≥25% compared with baseline^c^ and <LLN Increase in SCr or LDH level ≥25% compared with baseline^c^ and >ULN
Clinical signs and symptoms of TMA^d^	Clinical signs and symptoms of TMA considered definitely related to aHUS, including:
	Thrombosis Seizure Decreased renal function Proteinuria (new or worse compared with baseline and >1+ or >30 mg/dL) Haematuria (new or worse compared with baseline and >50 RBC/HPF) Increased haemolytic anaemia Biopsy-proven TMA Other (e.g. extrarenal TMA manifestations including confusion, cardiovascular abnormalities, pericarditis, gastrointestinal symptoms and diarrhoea)
Intervention^d^	The patient received PE/PI, dialysis, blood transfusions or renal transplant due to a TMA manifestation

a As determined by changes in laboratory parameters with ongoing follow-up.

b Measurements were required to be confirmed by a second measurement ≥28 days apart with no interruption.

c Baseline was defined for each on period as the last laboratory value during the preceding off period, and for each off period as the last value during the preceding on period.

d As determined at the discretion of the investigator.

HPF, high-powered field; LDH, lactate dehydrogenase; LLN, lower limit of normal; PE/PI, plasma exchange/plasma infusion; RBC, red blood cells; SCr, serum creatinine; ULN, upper limit of normal.

### Statistical methods

Time to first TMA manifestation was defined as time from the start of the current study (i.e. end of the parent study) to first TMA manifestation during the current study. Patients who did not have a TMA manifestation were censored at data cut-off or study discontinuation, whichever occurred first. Time to first TMA manifestation was analysed using Cox proportional hazards models with treatment status as a time-dependent explanatory variable and complement abnormality status as a covariate. Hazard ratios (HRs) and P values were obtained for comparisons off and on treatment and between identified and no identified complement abnormality subgroups.

## RESULTS

### Patients and exposure

**FIGURE 1:**
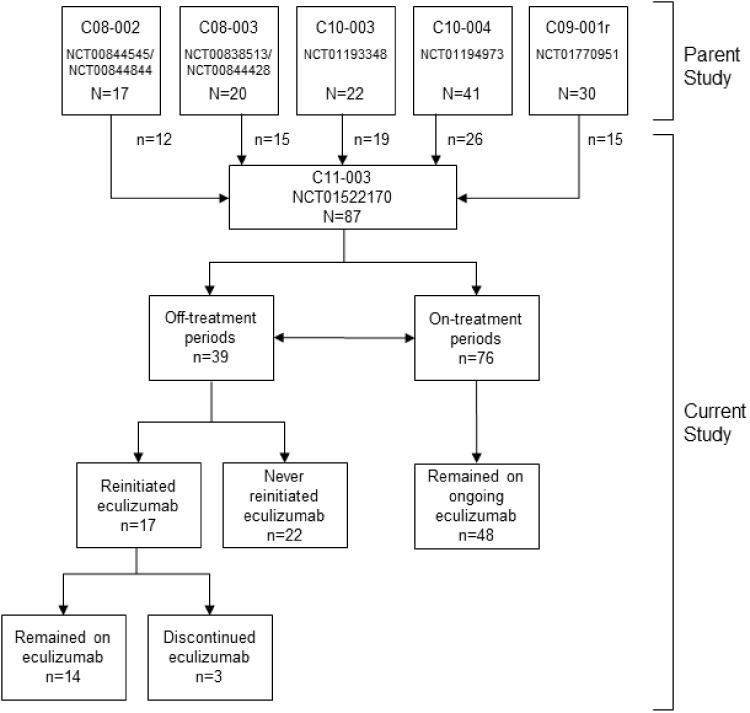
Patient disposition.

Overall, 130 patients were enrolled in the parent studies. By the data cut-off for this analysis, 87 patients had enrolled in the current study. Of these, 39 (45%) had off-treatment periods whereas 76 (87%) had on-treatment periods. Seventeen patients (44%) with off-treatment periods reinitiated eculizumab; of these, 14 (82%) remained on therapy once they reinitiated (Figure 1). Age, frequency of complement abnormalities and kidney transplant status were not different between patients with ongoing eculizumab therapy versus those who discontinued (Table 2). Twenty-two patients (25%) had renal transplants, including eight patients (21%) who discontinued eculizumab and 14 (29%) on ongoing eculizumab. A median (range) of 11.0 (0.0–230.0) plasma exchanges or plasma infusions were used by the overall population before eculizumab initiation, including 7.0 (0.0–64.0) in patients who discontinued eculizumab and 13.3 (0.0–230.0) in patients who never discontinued eculizumab. Dialysis was required by 29/87 patients (33%) before parent study enrolment, and use was more frequent in those who discontinued eculizumab (39%) compared with those who never discontinued eculizumab (29%). Including parent studies, patients had a total median (range) of 45.9 (1.3–86.9) months of eculizumab exposure. In the current study, median (range) follow-up was 20.1 (0.7–79.5) months off and 26.1 (0.7–64.2) months on treatment.

**Table 2.** Demographic and baseline clinical characteristics in the parent studies

Characteristic	Ongoing eculizumab	Discontinued	Total
	(*n* = 48)	(*n* = 39)	(*n* = 87)
Age at first-ever infusion of eculizumab, years			
Median (range)	24.0 (0.0–65.0)	21.0 (0.0–80.0)	22.0 (0.0–80.0)
<12, *n* (%)	13 (27)	11 (28)	24 (28)
12 to <18, *n* (%)	6 (13)	5 (13)	11 (13)
≥18, *n* (%)	29 (60)	23 (59)	52 (60)
Age at entry to current study, years			
Median (range)	27.5 (2.5–67.6)	25.1 (1.6–81.6)	26.0 (1.6–81.6)
<12, *n* (%)	10 (21)	11 (28)	21 (24)
12 to <18, *n* (%)	3 (6)	0 (0)	3 (3)
≥18, *n* (%)	35 (73)	28 (72)	63 (72)
Female, *n* (%)	30 (63)	23 (59)	53 (61)
Complement gene mutation or autoantibody, *n* (%)	31 (65)	20 (51)	51 (59)
*CFH*^a^	12 (25)	8 (21)	20 (23)
CFH autoantibodies^b^	6 (13)	1 (3)	7 (8)
*C3*	6 (13)	1 (3)	7 (8)
*CD46 (MCP)*	2 (4)	5 (13)	7 (8)
*CFI*	3 (6)	4 (10)	7 (8)
*C3*, *CFHR3-CFHR1*	1 (2)	0	1 (1)
*CD46 (MCP)*, *CFI*	1 (2)	0	1 (1)
*CFB*	0	1 (3)	1 (1)
Identified *CFHR1*, *CFHR3* deletion	0	1 (3)	1 (1)
Time from last pretreatment aHUS manifestation to first-ever dose of eculizumab, median (range), months	2.3 (0.0–47.4)	0.5 (0.0–19.2)	0.9 (0.0–47.4)
Family history of aHUS, *n* (%)	8 (17)	8 (21)	16 (18)
Time from aHUS diagnosis to first-ever dose of eculizumab, median (range), months	18.4 (0.0–313.3)	0.9 (0.0–178.1)	4.0 (0.0–313.3)
History of TMA manifestations, *n* (%)			
Single	29 (60)	27 (69)	56 (64)
Multiple	19 (40)	12 (31)	31 (36)
Prior renal transplant, *n* (%)	14 (29)	8 (21)	22 (25)
PE/PI sessions per patient, median (range)	13.3 (0.0–230.0)	7.0 (0.0–64.0)	11.0 (0.0–230.0)
Dialysis at baseline^c^, *n* (%)	14 (29)	15 (39)	29 (33)
eGFR^d^ at baseline, median (range), mL/min/1.73 m^2^	21.2 (8.4–128.3)	12.1 (5.3–105.5)	18.9 (5.3–128.3)

a Includes patients with additional abnormalities [i.e. *C3*, *CD46* (*MCP*), *CFI*, *CFHR1*, *CFHR3*, and *CFHR3-CFHR1*].

b Includes patients with additional abnormalities (i.e. *CFHR1, CFHR3*, *CFHR3-CFHR1*).

c Dialysis at baseline was defined as any dialysis that occurred within 7 days prior to or 14 days following the first eculizumab dose in the parent study.

d eGFR was defined as 10 mL/min/1.73 m^2^ when a patient was on dialysis.

PE/PI, plasma exchange/plasma infusion.

Compared with patients who continued on eculizumab, those who discontinued had a shorter interval from initial aHUS diagnosis, as well as the most recent pretreatment TMA manifestation, to first-ever eculizumab dose in the parent study. Patients who discontinued also presented with lower estimated glomerular filtration rates (eGFRs) and were more frequently dialysis dependent at initiation of eculizumab (Table 2).

Non-labelled eculizumab regimens were received by 33/87 patients (38%) during the current study. Of these, 11 always received non-labelled doses and 22 had periods of labelled and non-labelled regimens. Median (range) duration of therapy was 25.7 (0.5–60.4) months during labelled and 14.3 (0.4–64.3) months during non-labelled regimens. Dosing higher than labelled accounted for 0.4% of the total patient-years for non-labelled regimens.

### TMA manifestations

When using the per-protocol definition (Table 1), 28 TMA manifestations occurred. This included 14 TMA manifestations in 11/39 patients (28%) off treatment and 14 TMA manifestations in 10/76 patients (13%) on treatment (Tables 3 and 4). TMA manifestation rate per 100 patient-years was 19.9 off and 7.3 on treatment (63% lower; HR, 4.7; P = 0.0008; Table 5). Patients treated with non-labelled and labelled regimens had TMA manifestation rates of 12.1 and 5.2 per 100 patient-years, respectively (39% and 74% lower, respectively, versus off treatment).

**Table 3.** Reported TMA manifestations in patients off treatment with eculizumab

Patient demographics and clinical characteristics				Eculizumab therapy^a^					
Patient	Sex	Patient age	Complement abnormality	Eculizumab duration before discontinuation	Duration of discontinuation before TMA manifestation	TMA manifestation based on single lab criterion	Criteria achieved for TMA manifestation	SAE/ hospitalization^b^	Eculizumab reinitiation^c^
		(years)		(months)	(months)				
1	Male	9	*CFH*	6	1.3	No	↑SCr, ↑LDH	No	Yes
2	Male	11	*CFI*	5	17	No	↑SCr, ↑LDH, ↓platelets	Yes	Yes
				0.7	3.7	No	↑SCr, ↑LDH, ↓platelets, transfusion	Yes	Yes
3	Male	4	*CFH*	14	2.5	No	↓Platelets, ↓renal function, ↑haemolytic anaemia	No	Yes
4	Male	38	*CFH*	27	2.6	Yes	↑SCr	Yes	Yes
5	Female	25	*MCP*	37	5	No	↑SCr, ↑LDH, ↓Hb, ↓haptoglobin, renal impairment	Yes	Yes
6	Female	39	*CFI*	37	7	Yes	↓Platelets	Yes	Yes
7	Female	84	None identified	3	28	No	↑LDH, ↓platelets	Yes	No
				3	42	No	Signs of reactivation of TMA with clinical repercussion	No	No
8	Male	26	*MCP*	6	18	No	↑SCr, transfusion	Yes	No
				6	34	No	↑SCr, transfusion	Yes	Yes
9	Male	27	None identified	50	2	No	↓Haptoglobin	Yes	Yes
10	Female	73	CFH autoantibodies, *CFHR1-3*	34	17	Yes	↑SCr	No	No
11	Female	20	*CFH*, *C3*	6	2	No	↓Platelets, acute renal failure	No	Yes

a During the current study only (i.e. excluding the parent study).

b Before or during TMA manifestation.

c Seventeen of 39 patients (43.6%) reinitiated eculizumab after discontinuation of therapy. Median (range) time to reinitiation was 2.6 (0.7–69.3) months. Fourteen of 17 patients (82%) continue on therapy after reinitiation.

Hb, haemoglobin; LDH, lactate dehydrogenase; SCr, serum creatinine.

**Table 4.** Reported TMA manifestations in patients on treatment with eculizumab

Patient demographics and clinical characteristics				Eculizumab therapy^a^				
Patient	Sex	Patient age (years)	Complement abnormality	Treatment duration (months)	Labelled regimen^b^	TMA manifestation based on single lab criterion	Criteria achieved for TMA manifestation	SAE/ hospitalization^c^
1	Female	4	None identified	26	No	Yes	↑SCr	No
2	Female	31	*CFH*	28	Yes	Yes	↓Platelets	Yes
3	Female	44	*CFH*	20	Yes	Yes	↑LDH	No
4	Female	12	*CFH*	59	Yes	Yes	↑SCr	No
5	Female	44	*CFI*	19^d^	Yes	Yes	↓Platelets	Yes
6	Male	27	*MCP*	51	Yes	Yes	↑LDH	No
				60	No	Yes	↑LDH	No
7	Male	49	*CFI*	39	Yes	No	Dialysis	Yes
8	Female	10	*CFH*	56	No	Yes	↑SCr	No
				61	No	No	↑SCr, ↓platelets,	No
							↑Proteinuria	
9	Male	15	*CFH*	41	No	Yes	↓Platelets	No
				44	No	Yes	↑SCr	No
				53	No	Yes	↑SCr	No
10	Male	20	None identified	3	Yes	Yes	↑LDH	No

a During the current study only (i.e. excluding the parent study).

b No TMA manifestations occurred while patients received dosing higher than approved by regulatory authorities.

c Before or during TMA manifestation.

d The patient’s first on-treatment period was 19 months, followed by an off-treatment period of 31 months and another on-treatment period of 10 months.

LDH, lactate dehydrogenase; SCr, serum creatinine.

**Table 5.** TMA manifestation rates

	Eculizumab treatment status		Eculizumab dosing		Excluding single laboratory change criterion	
Parameter	Off treatment	On treatment	Non-labelled regimen	Labelled regimen	Off treatment	On treatment
	(*n* = 39)	(*n* = 76)	(*n* = 33)	(*n* = 65)	(*n* = 39)	(*n* = 76)
Patients with manifestation, *n* (%)	11 (28)	10 (13)	4 (12)	7 (11)	8 (21)	2 (3)
Total number of manifestations	14	14	7	7	11	2
Total patient-years	70.5	192.8	57.9	135.0	70.5	192.8
TMA manifestation rate/ 100 patient-years	19.9	7.3	12.1	5.2	15.6	1.0
Fold change in rate^a^	2.7	Ref	2.3	Ref	15.6	Ref
Per cent change compared with off treatment^b^ (%)	Ref	−63	−39	−74	Ref	−94
HR (P value)^c^	4.7 (P = 0.0008)	Ref	1.3 (P = 0.7000)^d^	Ref	16.8 (P = 0.0010)	Ref

a Off treatment compared with on treatment (overall) or non-labelled compared with labelled regimen for the same analysis.

b On treatment (overall), non-labelled or labelled regimen compared with off treatment for the same analysis.

c HRs were based on Cox proportional hazards model of time to first TMA manifestation, with treatment status as a time-dependent explanatory variable and complement abnormality status as a covariate.

d Compared with the labelled dosing regimen of eculizumab.

Ref, reference value.

### Characteristics of TMA manifestations

TMA manifestations off treatment were more frequently associated with multiple laboratory criteria for TMA, clinical signs and symptoms of TMA, interventions, SAEs and/or hospitalizations (Tables 3 and 4). Eleven of 14 TMA manifestations (79%) in patients off treatment included multiple laboratory TMA criteria and/or an intervention, compared with 2/14 TMA manifestations (14%) on treatment. Patients required hospitalization during 9/14 TMA manifestations (64%) off treatment and 3/14 TMA manifestations (21%) on treatment.

### TMA manifestation rate excluding TMAs based on single laboratory value changes

An abnormality in a single laboratory value may not be considered as TMA clinically; therefore, a *post hoc* analysis was performed to better reflect TMA evaluation in clinical practice. TMA defined by a change from baseline in a single laboratory value occurred in 3/14 TMA manifestations (21%) off treatment and 12/14 TMA manifestations (86%) on treatment (Tables 3 and 4). When using this definition, 11 TMA manifestations occurred in 8/39 patients (21%) off treatment and two TMA manifestations occurred in 2/76 patients (3%) on treatment. The TMA manifestation rate per 100 patient-years was 15.6 off and 1.0 on treatment (94% decrease; HR, 16.8; P = 0.0010) (Table 5).

### TMA manifestations by complement abnormality status

The majority of patients who reported TMA manifestations as defined per protocol had complement abnormalities (Tables 3 and 4), particularly in those with *CFH* [10/17 (59%)] and *CFI* mutations [4/17 (24%)]. Rates were higher for patients with identified complement abnormalities compared with no identified complement abnormalities (HR, 4.5; P = 0.0082).

### Safety

Overall, treatment with eculizumab was well tolerated. The occurrence of serious targeted TEAEs during the current study was similar off and on treatment (Table 6). Two patients from the parent retrospective study reported meningococcal infections during the current study; both were determined to be probably related to eculizumab. Both patients were treated and recovered while continuing eculizumab on schedule. Diagnoses/underlying conditions associated with reported serious targeted TEAEs of renal impairment that did not meet criteria for TMA included new kidney transplant, renal graft rejection, multiorgan failure, dehydration, infection and interstitial tubulopathy. One adult patient, who received non-labelled dosing during the current study, died due to severe intensive care complications and severe multiorgan dysfunction after gastrointestinal haemorrhage, lithiasic cholecystitis and severe sepsis, which were determined to be unrelated to eculizumab.

**Table 6.** Serious targeted TEAEs reported in the current study

TEAE	Off treatment^a^	On treatment
	(*n* = 39)	(*n* = 76)
Any serious targeted TEAEs, rate per 100 patient-years	15.6 (11)	14.0 (27)
Renal impairment^b^	11.3 (8)	8.3 (16)
Infection, other	2.8 (2)	2.1 (4)
Infection, septic	1.4 (1)	2.1 (4)
Infection, meningococcal	0.0 (0)	1.0 (2)
Leucopaenia	0.0 (0)	0.5 (1)

Data are presented as *n* (%).

a Data were obtained retrospectively between the end of the parent study and enrolment in the current study for patients off treatment and on treatment.

b TEAEs of renal impairment were evaluated and reported by each investigator, and no set definition was used.

## DISCUSSION

Results from this interim analysis of a non-randomized, prospective observational study demonstrate that rates of TMA manifestations in patients with aHUS were 2.7-fold higher off compared with on eculizumab (63% lower), despite longer follow-up on treatment. TMA manifestation rates were lowest during labelled dosing regimens (74% lower than off treatment), higher during non-labelled regimens (39% lower than off treatment) and highest off treatment.

The per-protocol definition of TMA manifestations was broad, including changes in laboratory values, clinical signs and symptoms of TMA related to aHUS, and/or interventions related to TMA. Thus, reported TMA per this definition represented varying degrees of clinical deterioration. Importantly, there is no single, agreed-upon definition of TMA based upon a single laboratory value in clinical practice. TMA manifestations off treatment were associated with multiple laboratory criteria, clinical sequelae (e.g. renal impairment and acute renal failure), SAEs, hospitalizations and/or required interventions (e.g. transfusion) in 13/14 cases (93%). In contrast, TMA manifestations on treatment typically comprised changes in single laboratory values with no clinical signs/symptoms. Therefore, *post hoc* analyses were conducted to provide insights using a more stringent TMA definition that we believe more closely defines TMA in the setting of aHUS. When TMA manifestations based only on changes in single laboratory values were excluded, the rate off treatment was 15.6-fold higher than on treatment. These results taken together could suggest worse outcomes for patients who discontinue eculizumab, although it is possible that changes in single laboratory values may signal subclinical disease processes.

Patients with identified complement abnormalities had statistically significantly higher TMA rates than patients with no identified abnormalities. *CFH* and *CFI* mutations were predominant among patients who experienced TMA, regardless of treatment status. This finding is consistent with previous observational studies of the natural history of aHUS [2, 3]. In a small observational study, Ardissino *et al.* [28, 29] also noted particular risk for TMA in patients who chose to discontinue eculizumab with *CFH* mutations, and *CFI* mutations to a lesser extent. In a retrospective study of eculizumab discontinuation in a French cohort (*n* = 38) [27], all 12 patients (32%) with TMA post-eculizumab discontinuation had rare or novel *CFH* or *MCP* variants; both were independent risk factors for TMA following discontinuation. However, patients with transplant, on chronic dialysis or ‘secondary’ aHUS were excluded from the French cohort. Results of the current analysis, which are from the single largest prospective study of TMA risk following eculizumab discontinuation, reinforce previous findings that patients who discontinued eculizumab were at greater risk for TMA compared with patients on treatment, and particularly those with identified complement abnormalities. In this study, patients without identified abnormalities were at a significantly lower yet distinct risk for TMA after eculizumab discontinuation. However, genetic analyses were performed during the parent studies several years ago; thus, it is possible that novel mutations not known at that time have been left unrecognized.

Overall, characteristics of patients with TMA manifestations were highly heterogeneous with respect to age, treatment duration and duration of eculizumab before onset of TMA. Age, frequency of complement abnormalities and kidney transplant status did not differ between patients who discontinued versus remained on eculizumab. However, patients who discontinued appeared to have poorer renal function at baseline in the parent study and initiated eculizumab more rapidly. Such patients may have had clinically significant renal improvement with eculizumab, since they initiated treatment in a rapid manner [31], followed by the clinical decision to discontinue treatment after recovery. Discontinuation of therapy was not randomized, potentially allowing selection bias for continuing versus discontinuing eculizumab. In the previously cited studies [26–28], not all patients with aHUS were included. In the current study, all patients who met inclusion criteria for the parent studies (including those with dialysis and renal transplants) were allowed to enrol. Further studies are needed to identify patient characteristics potentially associated with increased risk for TMA after eculizumab discontinuation.

Notably, 17/39 patients (44%) reinitiated eculizumab following a period of discontinuation, including 9/11 patients (82%) who had TMA manifestations while off treatment. After reinitiation, 14/17 patients (82%) continued eculizumab. Longer term evaluation may provide additional insight as to clinical outcomes associated with therapy stops and restarts.

Collectively, the current results reinforce the need for ongoing treatment with eculizumab to minimize risk of TMA in patients with aHUS, particularly those with an identified complement abnormality. Although thorough genetic testing informs prognosis, additional considerations when optimizing treatment strategy include the patient’s unique clinical situation, age, TMA and family histories, as well as recognition of the complex and unpredictable nature of aHUS. For individual patients in whom discontinuation of eculizumab is being considered, clinicians would be well advised to consult an expert centre in the field while ensuring that the patient: (i) has been treated for a sufficiently long period to ensure maximal organ function recovery; (ii) can be monitored closely for signs and/or symptoms of TMA; and (iii) has immediate access to eculizumab so treatment can be restarted at the first signs and/or symptoms of TMA [27, 32].

As was observed in the parent studies [7–10], eculizumab was generally well tolerated in the current study. Rates of serious targeted TEAEs, including infections, reported off and on treatment were similar. In particular, rates of renal impairment were relatively high both off and on treatment, but commonly associated with new kidney transplantation and existing graft failure. Two patients reported meningococcal infections during the current study. Both recovered and there were no changes in eculizumab dosing. Frequency of meningococcal infections [2/87 patients (2%)] is similar to that from the overall parent trial programme of eculizumab in aHUS [two cases/100 total patients (2%)] [7–10]. Overall, the reported meningococcal infection rate in patients treated with eculizumab is 0.3 events/100 patient-years [33]. Regulatory guidance for eculizumab includes increased susceptibility to meningococcal infection [5, 6]. Patients should be counselled in order to fully understand potential benefits and risks of treatment, early signs of meningococcal disease and processes for seeking immediate medical care. Risks of potentially severe complications, including meningococcal infection, should be considered during the decision-making process regarding initiating treatment or discontinuing eculizumab. Long-term evaluations of the eculizumab safety profile will be included in future analyses from the Global aHUS Registry [34].

An important study limitation was its open-label and observational nature. Voluntary patient enrolment into this prospective study may have introduced selection bias because data are not available for patients who completed a parent study but did not consent to enrolment in the current study. In this analysis, 43/130 patients (33%) who enrolled in one of the parent studies had not continued into the current study. During the parent and current studies, which together included a median exposure of 45.9 months, withdrawal of eculizumab due to an AE was uncommon. One adult patient died due to multiorgan failure following a reduced dosing regimen. One paediatric patient discontinued eculizumab in the parent study due to agitation [9]. Three patients with previous renal transplants and poor renal function (eGFR <30 mL/min/1.73 m^2^) at the start of treatment discontinued eculizumab following reports of renal impairment in the current study; of these, one later restarted eculizumab and the other two received additional renal transplants. Additional studies are needed to further understand patient and physician rationale for discontinuing and reinitiating treatment.

Taken together, findings from this interim analysis suggest that patients with aHUS have an increased risk for TMA manifestations after discontinuation of eculizumab or during non-labelled regimens compared with labelled eculizumab dosing. These results support current regulatory guidance [5, 6] in noting potential risk for TMA following discontinuation of eculizumab. Evidence demonstrates that patients had a 63–94% lower risk of TMA on eculizumab therapy, depending on the definition used. Future analyses will allow for further characterization of TMA and evaluation of longer term outcomes on labelled or non-labelled regimens of eculizumab and after therapy discontinuation.

## References

[1] Noris M, Remuzzi G. Atypical hemolytic-uremic syndrome. N Engl J Med 2009; 361: 1676–1687 19846853 10.1056/NEJMra0902814

[2] Noris M, Caprioli J, Bresin E et al Relative role of genetic complement abnormalities in sporadic and familial aHUS and their impact on clinical phenotype. Clin J Am Soc Nephrol 2010; 5: 1844–1859 20595690 10.2215/CJN.02210310 PMC2974386

[3] Fremeaux-Bacchi V, Fakhouri F, Garnier A et al Genetics and outcome of atypical hemolytic uremic syndrome: a nationwide French series comparing children and adults. Clin J Am Soc Nephrol 2013; 8: 554–562 23307876 10.2215/CJN.04760512 PMC3613948

[4] Noris M, Mescia F, Remuzzi G. STEC-HUS, atypical HUS and TTP are all diseases of complement activation. Nat Rev Nephrol 2012; 8: 622–633 22986360 10.1038/nrneph.2012.195

[5] US Food and Drug Administration. Soliris (eculizumab) [Prescribing Information]. New Haven, CT: Alexion Pharmaceuticals, Inc, 2017

[6] European Medicines Agency. Soliris (eculizumab) [Summary of Product Characteristics]. Paris, France: Alexion Europe SAS, 2017

[7] Legendre CM, Licht C, Muus P et al Terminal complement inhibitor eculizumab in atypical hemolytic-uremic syndrome. N Engl J Med 2013; 368: 2169–2181 23738544 10.1056/NEJMoa1208981

[8] Licht C, Greenbaum LA, Muus P et al Efficacy and safety of eculizumab in atypical hemolytic uremic syndrome from 2-year extensions of phase 2 studies. Kidney Int 2015; 87: 1061–1073 25651368 10.1038/ki.2014.423 PMC4424817

[9] Greenbaum LA, Fila M, Ardissino G et al Eculizumab is a safe and effective treatment in pediatric patients with atypical hemolytic uremic syndrome. Kidney Int 2016; 89: 701–711 26880462 10.1016/j.kint.2015.11.026

[10] Fakhouri F, Hourmant M, Campistol JM et al Terminal complement inhibitor eculizumab in adult patients with atypical hemolytic uremic syndrome: a single-arm, open-label trial. Am J Kidney Dis 2016; 68: 84–93 27012908 10.1053/j.ajkd.2015.12.034

[11] Vilalta R, Al-Akash S, Davin J et al Eculizumab therapy for pediatric patients with atypical hemolytic uremic syndrome: efficacy and safety outcomes of a retrospective study [abstract 1155]. Haematologica 2012; 97 (Suppl 1): 47922492290

[12] Chatelet V, Fremeaux-Bacchi V, Lobbedez T et al Safety and long-term efficacy of eculizumab in a renal transplant patient with recurrent atypical hemolytic-uremic syndrome. Am J Transplant 2009; 9: 2644–2645 19775316 10.1111/j.1600-6143.2009.02817.x

[13] Alachkar N, Bagnasco SM, Montgomery RA. Eculizumab for the treatment of two recurrences of atypical hemolytic uremic syndrome in a kidney allograft. Transpl Int 2012; 25: e93–e95 22591029 10.1111/j.1432-2277.2012.01497.x

[14] Cayci FS, Cakar N, Hancer VS et al Eculizumab therapy in a child with hemolytic uremic syndrome and CFI mutation. Pediatr Nephrol 2012; 27: 2327–2331 22903728 10.1007/s00467-012-2283-9

[15] Carr R, Cataland SR. Relapse of aHUS after discontinuation of therapy with eculizumab in a patient with aHUS and factor H mutation. Ann Hematol 2013; 92: 845–846 23154867 10.1007/s00277-012-1622-z

[16] Gulleroglu K, Fidan K, Hancer VS et al Neurologic involvement in atypical hemolytic uremic syndrome and successful treatment with eculizumab. Pediatr Nephrol 2013; 28: 827–830 23389237 10.1007/s00467-013-2416-9

[17] Pu JJ, Sido A. Successful discontinuation of eculizumab therapy in a patient with aHUS. Ann Hematol 2014; 93: 1423–1425 24346709 10.1007/s00277-013-1972-1

[18] Coppo R, Peruzzi L, Amore A et al Dramatic effects of eculizumab in a child with diffuse proliferative lupus nephritis resistant to conventional therapy. Pediatr Nephrol 2015; 30: 167–172 25173358 10.1007/s00467-014-2944-y

[19] Habbig S, Bergmann C, Weber LT. Discontinuation of eculizumab in a patient with atypical hemolytic uremic syndrome due to a mutation in *CFH*. Am J Kidney Dis 2016; 67: 532–533 10.1053/j.ajkd.2015.11.009 26724167

[20] Hayes W, Tschumi S, Ling SC et al Eculizumab hepatotoxicity in pediatric aHUS. Pediatr Nephrol 2015; 30: 775–781 25416628 10.1007/s00467-014-2990-5

[21] Java A, Edwards A, Rossi A et al Cytomegalovirus-induced thrombotic microangiopathy after renal transplant successfully treated with eculizumab: case report and review of the literature. Transpl Int 2015; 28: 1121–1125 25864519 10.1111/tri.12582 PMC4529787

[22] Canigral C, Moscardo F, Castro C et al Eculizumab for the treatment of pregnancy-related atypical hemolytic uremic syndrome. Ann Hematol 2014; 93: 1421–1422 24306089 10.1007/s00277-013-1970-3

[23] De Sousa Amorim E, Blasco M, Quintana L et al Eculizumab in pregnancy-associated atypical hemolytic uremic syndrome: insights for optimizing management. J Nephrol 2015; 28: 641–645 25712233 10.1007/s40620-015-0173-5

[24] Wetzels JF, van de Kar NC. Discontinuation of eculizumab maintenance treatment for atypical hemolytic uremic syndrome. Am J Kidney Dis 2015; 65: 342 10.1053/j.ajkd.2014.04.039 25616634

[25] Toyoda H, Wada H, Miyata T et al Disease recurrence after early discontinuation of eculizumab in a patient with atypical hemolytic uremic syndrome with complement C3 I1157T mutation. J Pediatr Hematol Oncol 2016; 38: e137–e139 26840081 10.1097/MPH.0000000000000505

[26] Sheerin NS, Kavanagh D, Goodship TH et al A national specialized service in England for atypical haemolytic uraemic syndrome-the first year’s experience. QJM 2016; 109: 27–33 25899302 10.1093/qjmed/hcv082

[27] Fakhouri F, Fila M, Provôt F et al Pathogenic variants in complement genes and risk of atypical hemolytic uremic syndrome relapse after eculizumab discontinuation. Clin J Am Soc Nephrol 2017; 12: 50–59 27799617 10.2215/CJN.06440616 PMC5220663

[28] Ardissino G, Testa S, Possenti I et al Discontinuation of eculizumab maintenance treatment for atypical hemolytic uremic syndrome: a report of 10 cases. Am J Kidney Dis 2014; 64: 633–637 24656451 10.1053/j.ajkd.2014.01.434

[29] Ardissino G, Possenti I, Tel F et al Discontinuation of eculizumab treatment in atypical hemolytic uremic syndrome: an update. Am J Kidney Dis 2015; 66: 172–173 10.1053/j.ajkd.2015.04.010 26111906

[30] Macia M, de Alvaro Moreno F, Dutt T et al Current evidence on the discontinuation of eculizumab in patients with atypical haemolytic uraemic syndrome. Clin Kidney J 2017; 10: 310–319 28621343 10.1093/ckj/sfw115 PMC5466111

[31] Vande Walle J, Delmas Y, Ardissino G et al Improved renal recovery in patients with atypical hemolytic uremic syndrome following rapid initiation of eculizumab treatment. J Nephrol 2017; 30: 127–134 26995002 10.1007/s40620-016-0288-3 PMC5316393

[32] Loirat C, Fakhouri F, Ariceta G et al An international consensus approach to the management of atypical hemolytic uremic syndrome in children. Pediatr Nephrol 2016; 31: 15–39 25859752 10.1007/s00467-015-3076-8

[33] Drug Safety and Risk Management Advisory Committee. Briefing Document for Soliris^®^ (eculizumab*).* Cheshire, CT: Alexion Pharmaceuticals, Inc, 2014

[34] Licht C, Ardissino G, Ariceta G et al The global aHUS registry: methodology and initial patient characteristics. BMC Nephrol 2015; 16: 207 26654630 10.1186/s12882-015-0195-1 PMC4674928

